# Multicenter Study of Tetanus Patients in Fujian Province of China: A Retrospective Review of 95 Cases

**DOI:** 10.1155/2020/8508547

**Published:** 2020-03-15

**Authors:** Xiaojing Wang, Rongguo Yu, Xiuling Shang, Jun Li, Ling Gu, Richun Rao, Xianliang Han, Mingzhi Chen, Jian Yang, Jiafang Wu, Huilong Chen, Hongyu Huang

**Affiliations:** ^1^Department of Critical Care Medicine, Fujian Provincial Hospital, Fujian Provincial Center for Critical Care Medicine, Fujian Medical University, Fuzhou, Fujian 350001, China; ^2^Department of Critical Care Medicine, Mindong Hospital of Ningde City, Ningde, Fujian 355000, China; ^3^Department of Infectious Diseases, Chaoyang District of Affiliated Zhangzhou Municipal Hospital of Fujian Medical University, Zhangzhou, Fujian 363000, China; ^4^Department of Critical Care Medicine, Jinjiang Municipal Hospital, Jinjiang, Fujian 362200, China; ^5^Department of Critical Care Medicine, Affiliated Nanping First Hospital of Fujian Medical University, Nanping, Fujian 353000, China

## Abstract

**Background:**

Tetanus is a life-threatening disease in developing countries and is accompanied by a high mortality rate. Although China is the world's largest developing country, there have been few clinical studies on tetanus in China. The purpose of this study was to investigate the epidemiology, incidence, and management of tetanus in Fujian Province and to understand the current treatment and prognosis of tetanus patients.

**Methods:**

This was a retrospective, multicenter observational study of patients who presented with a clinical diagnosis of tetanus at 5 general hospitals in Fujian from January 2008 to December 2018. Data were analyzed using a computer software system.

**Results:**

A total of 95 patients were recruited, including 6 newborns. The average age of the adult tetanus patients was 55.53 ± 15.39 years old. None of the patients knew their previous history of tetanus immunization. The rate of having received human tetanus immunoglobulin (HTIG) was 9.47%. A total of 73 (76.84%) patients were cured, 17 (17.89%) patients had an unknown prognosis, and 5 (5.26%) patients died. Age, severity of illness, and complications all increased the total duration of hospitalization. Compared with endotracheal intubation, tracheotomy increased the length of stay in the ICU (Intensive Care Unit) but did not affect the total hospital length of stay for mechanical ventilation.

**Conclusions:**

With the promotion of nationwide immunization against tetanus and the development of critical care medicine, morbidity and mortality rates of tetanus in Fujian are low. It is important to increase awareness among local physicians and staff in charge of tetanus immunization programs and with regard to neonatal tetanus and drug-induced tetanus. The prevention and treatment of tetanus in developing countries should arouse widespread concern in society.

## 1. Introduction

Tetanus is still a common disease in developing countries and has a high mortality rate [1]. Although the incidence of tetanus in developed countries is low and the prognosis good, the medical burden of tetanus remains high [2].

The pathogen causing tetanus is *Clostridium tetani*, a gram-positive anaerobic bacterium that exists widely in nature in the form of spores. Following contact with damaged skin and mucous membranes, the spores enter the body and become endospores in the local anaerobic environment, producing and releasing a large amount of tetanus toxin [3]. The most common presenting symptom of tetanus is trismus, and the disease is diagnosed primarily through clinical presentation rather than through bacteriological culture [4, 5].

As a vaccine-preventable disease, the morbidity and mortality rates of tetanus can be significantly reduced in intensive care facilities and well-staffed medical settings. However, there has been little clinical research on the present status of tetanus in China. Hence, this study is aimed at retrospectively analyzing the clinical data of tetanus patients and at understanding the current treatment and prognosis status of tetanus patients in Fujian.

## 2. Materials and Methods

### 2.1. Study Setting and Design

This was a multicenter retrospective study. Data were obtained from 5 hospitals in the Fujian region from January 2008 to December 2018.

### 2.2. Study Object

This study included all patients of all age groups and both sexes with a clinical diagnosis of tetanus. The diagnosis of tetanus was entirely clinical and based on the presence of one or more of the following: (1) trismus, (2) rigidity of the neck and/or abdomen, and (3) reflex spasms [4].

Rigidity and/or spasm limited to a wound-bearing area of the body was defined as localized tetanus; trismus and generalized rigidity with or without generalized spasm were defined as generalized tetanus. Tetanus occurring during the neonatal period was classified as neonatal tetanus. A form of localized tetanus restricted to the head and neck was classified as cephalic tetanus [4]. The tetanus severity was classified using the system reported by Ablett, which includes mild, moderate, severe, and very severe categories [6]. The incubation period was defined as the period from the onset of injury to the onset of first symptoms. Treatment for tetanus includes elimination of tetanus toxin, neutralization of circulating toxins, and related supportive treatment [4]. Tetanus antitoxin (TAT) is a liquid antitoxin globulin preparation from plasma obtained by immunizing horses with tetanus toxoid. Human tetanus immunoglobulin (HTIG) is a human immunoglobulin separated and purified from plasma obtained from those who received a tetanus vaccine. All clinical data were obtained from medical records.

### 2.3. Statistical Analysis

Statistical analysis was performed by using the statistical package for the social sciences (SPSS) version 25.0 for Windows (SPSS, Chicago, IL, USA). The mean ± standard deviation (SD), median, and range were calculated for continuous variables, whereas proportions and frequency tables were used to summarize categorical variables. Analysis of variance was applied for continuous measurement data that were normally distributed, whereas nonparametric tests were used for nonnormally distributed data. The threshold for significance was *P* < 0.05.

### 2.4. Ethical Considerations

Ethical approval to conduct the study was granted by the ethics committee.

## 3. Results

### 3.1. Demographic Data

A total of 95 tetanus patients were enrolled in the study. The mean age of the 88 adult patients was 55.53 ± 15.39 years. The incubation period was not available for 3 drug users and 9 patients with unknown routes of infection. The remaining 83 patients had an average incubation period of 9 [4, 7] days. Other demographic data are shown in Table 1.

### 3.2. Clinical Features

Acute injury was the most common portal of entry. All neonatal tetanus infection injury routes were unclean deliveries. All three patients with drug addiction were classified as having severe tetanus or above. The most common clinical classification of tetanus was generalized, and all patients with localized tetanus had mild severity. More than half of the patients had autonomic nervous dysfunction and complications. Other clinical feature data are shown in Tables 2 and 3.

### 3.3. Treatment of the Patients

Two patients were discharged automatically without any treatment after diagnosis.

Seven patients were allergic to TAT. Only 9 patients received HTIG (9.47%). For 75 patients with hospital stays longer than 3 days, the TAT dose was 30,000 (30,000-52,500) UI/d. The TAT usage duration during the total length of hospital stay was 0.75 (0.288, 1.00), and 15 patients received TAT treatment during the entire hospital stay. Three patients did not receive antibiotics. The routine antibiotic regimen was penicillin or cephalosporin combined with nidazole. In the case of severe infection and sepsis, antibiotics were upgraded.

Six patients did not receive sedation. When the muscle rigidity and spasm of the whole body were mild, the commonly invoked plan was a combined intramuscular injection of diazepam and phenobarbital. When the symptoms were severe, diazepam was administered continuously via a micropump. For mechanical ventilation, sedation regimens included diazepam, phenytoin sodium, midazolam, and propofol.

A total of 39 patients were hospitalized for more than 3 days after an artificial airway was established, including 15 with endotracheal intubation (39.47%) and 24 with tracheotomy (60.53%). The duration of endotracheal intubation was 14.93 ± 9.59 days, and the duration of tracheotomy was 19 (14, 22.75) days. Thirty patients received mechanical ventilation. All patients with endotracheal intubation received mechanical ventilation, whereas 9 patients in the tracheotomy group did not receive mechanical ventilation. The total mechanical ventilation duration was 15 (9.75, 22) days, the mechanical ventilation duration for endotracheal intubation was 18 (4, 22) days, and the mechanical ventilation duration for tracheotomy was 15 (11, 23) days. There was no significant difference among them (*P* = 0.480).

### 3.4. Outcomes of Tetanus Patients

A total of 73 patients (76.84%) recovered, 5 (5.26%) died, and 17 (17.89%) had an unknown prognosis. The patients who died had severe or very severe conditions, including three with drug use, one following an electrical injury, and one following an acute injury; all mortality was caused by organ failure.

The total length of hospitalization ranged from 1 to 73 days; 13 patients had a length of hospitalization less than 3 days. The neonatal length of stay was 1 to 4 days, and none of them were admitted to the ICU. The total length of hospitalization of 78 adult patients was 23.81 ± 15.01 days. Among them, 60 patients were admitted to the ICU for treatment (76.92%), and the length of stay in the ICU was 24.85 ± 12.76 days.

There was a linear relationship between age and both total length of stay and length of stay in the ICU, with *P* values of 0.002 and 0.008 and correlation coefficients of 0.350 and 0.339, respectively.

Seventy-eight adult patients with a total length of hospitalization greater than 3 days were analyzed, and factors influencing the total length of hospitalization were inferred according to the ANOVA (Table 4). Additionally, 60 patients admitted to the ICU were analyzed, and the factors influencing the length of stay in the ICU were inferred according to the ANOVA (Table 5).

## 4. Discussion

In our study, the average age of the patients was 55 years old, and most of them were born before 1978. China's planned immunization program began in 1978, so most of the participants had not been immunized with tetanus vaccine. What is more, neither the history of reimmunization nor the history of immune strengthening was known for most patients in our study, and a research showed that screening for tetanus antibodies in their participants indicated an antibody positivity rate of only 47.62% [8]. Therefore, it is necessary to improve tetanus immunity among adults or posttraumatic patients as well as groups with an unknown early immunization history to further reduce the incidence of tetanus [2, 9].

With the improvement of medical conditions and the popularization of ICU wards, the death rate of neonatal tetanus in China was 222 cases by 2015, approximately 97.46% lower than the 13,572 cases in 1990 [1]. In this study, there were 6 neonatal patients, and all of these cases were associated with an unclean delivery and with an unknown prognosis. Although the government implements various monitoring and preventive measures to eliminate neonatal tetanus [10], in some remote areas, pregnant women still receive traditional delivery from institutions outside the hospital, leading to neonatal tetanus infection. Moreover, all 6 neonates in this study were discharged automatically without standard treatment, which requires extensive attention from society.

In this study, 3 patients with drug addiction all died. Because of unclean injections and the propensity of HIV infection, which reduces the titer of tetanus immune protection [11–13], tetanus is more likely to occur in individuals with drug addiction. Moreover, due to the adverse effects of drug abuse on the whole body, tetanus infection is often more serious, and its clinical manifestations are more complex. With the increasing number of individuals addicted to drugs in China, drug-induced tetanus cases in the clinic will continue to appear, and more attention should be given to these cases.

The use of tetanus antitoxin remains a specific therapy designed to neutralize the tetanus toxin. Nonuse or use of low-dose antitoxin was associated with high mortality [14]. However, TAT-induced allergic reactions can be as high as 5%~30% [7, 15]. In the present study, one patient was allergic to TAT; therefore, neither TAT nor HTIG (it cannot be obtained in some areas) preventive treatment was given after injury, leading to secondary infection of tetanus. In addition, the use of TAT is only effective when tetanus toxin is not combined with nerve tissue in the early stage of the disease; accordingly, there is no reason for continuous or increased use of TAT, which may even increase the risk of allergy and serum diseases [16]. At present, there is no clearly recommended route for tetanus administration, and the dose and duration of administration vary greatly among regions [2]. In this study, a TAT administration time of more than half of the total length of hospital stay occurred for more than half of the patients. It is necessary to further standardize treatment with TAT. Only 9 patients in our study received HTIG treatment, and the proportion was much lower than that in other countries [4, 17, 18]. Although it is believed that the use of human or equine antitoxins (TAT and HTIG) does not change the mortality rate of tetanus, HTIG has a longer half-life in the body and causes fewer adverse reactions [14]. It is necessary to promote HTIG treatment in clinical practice.

The hospitalization time of the patients with complications in our study tended to increase, and other studies have found that patients with concurrent infection had a lower survival at 90 days without mechanical ventilation [19]. Approximately 40% of patients with artificial airways were intubated; 60% received tracheotomy; in addition, approximately one-third of patients receiving tracheotomy did not receive mechanical ventilation. Although the total length of stay in the ICU in patients with tracheotomy was longer than that of patients with endotracheal intubation, there was no difference between the mechanical ventilation duration and total length of hospitalization. Current studies suggest that tracheotomy is superior to endotracheal intubation because it can reduce the incidence of tracheal stenosis caused by a lengthy period of endotracheal intubation [19].

By 2015, approximately 56,000 people worldwide have died due to tetanus, with a mortality rate in South Asia and sub-Saharan Africa of 79% [1]. However, the mortality rate of tetanus in developed countries is relatively low. Among the 499 patients admitted to tetanus in Japan from 2010 to 2016, only 34 (7%) died [20]. With the establishment and development of critical care medicine in China, the mortality rate of tetanus in 2015 had decreased by approximately 93.54% compared with that in 1990 [1]. In our study, only 5 adults (5.26%) died from tetanus, which was basically the same as the mortality rate in developed countries and significantly lower than that in other developing countries [1, 4, 17]. Nonetheless, the small number of patients included in this study cannot well reflect the current situation of tetanus in China, and more clinical studies should be performed in the future.

## 5. Conclusions

The mortality rate of tetanus patients in Fujian Province was low. Neonatal tetanus still occurs from time to time, and most of these patients do not receive standard treatment, which is a cause for social concern. With the increasing number of individuals addicted to drugs in recent years, tetanus caused by drug abuse needs to be given more attention. The average age of tetanus patients was 55 years old, and this group of the population has not received national planned immunizations. Therefore, the tetanus incidence can be better reduced by perfecting and strengthening the immunization program in the entire population, and a more standardized treatment regimen should be established.

## Figures and Tables

**Table 1:** Demographic data.

Variable	Number of patients	Percentages
Sex		
Male	63	66.32%
Female	32	33.68%
Age group (years)		
≤28 days	6	6.32%
1~17	1	1.05%
18~45	19	20.00%
46~65	46	48.42%
66~79	18	18.95%
>79	5	5.26%
Occupation		
Farmers	47	49.47%
Unemployed	14	14.74%
Others^∗^	34	35.79%
	95	100.00%

^∗^Others included labor/industrial workers, the retired people, and the newborn.

**Table 2:** Clinical features.

Variable	Number of patients	Percentages
Portals of entry		
Acute injury (prick, puncture, laceration, etc.)	72	75.79%
Injected drugs	3	3.16%
Unclean delivery	6	6.32%
Others^∗^	5	5.26%
Unknown	9	9.47%
Clinical classification		
Generalized	83	87.37%
Localized	4	4.21%
Cephalic	2	2.11%
Neonatal	6	6.32%
Severity of tetanus		
Mild	26	27.37%
Moderate	21	22.11%
Severe	40	42.11%
Very severe	8	8.42%
	95	100.00%

^∗^Others included medical interventions, animal bites, and electrical injuries.

**Table 3:** Clinical presentations and complications.

Clinical presentations and complications	Number of patients	Percentages
Clinical manifestation		
Body stiffness	69	72.63%
Body spasm	71	74.74%
Urinary retention	10	10.53%
Dysphagia	38	40.00%
Anhelation	31	32.63%
Fever	41	43.16%
Body aches	25	26.32%
Autonomic nervous dysfunction		
Yes	51	53.68%
No	44	46.32%
Complications		
No	35	36.84%
Yes	60	63.16%
Infection	52	86.67%
Pulmonary infections	45	86.54%
Urinary infection	9	17.31%
Others^∗^	7	13.46%
Airway obstruction	35	58.33%
Organ dysfunction	19	31.67%
Sepsis	13	21.67%
Digestive tract hemorrhage	6	10.00%
Others^#^	14	23.33%

^∗^Other sites of infection included wound infections, catheter-related blood stream infections, oral mucosal infections, and unknown source of infection. ^#^Other complications included acute respiratory distress syndrome and pulmonary embolism pneumothorax, hepatic dysfunction, myocardial injury, ischemic hypoxic cerebropathy, and pulmonary embolism.

**Table 4:** The factors influencing the total length of hospitalization.

Variable	Total length of hospitalization (days)	*P* value
Sex		
Male	21.18 ± 14.99	0.038
Female	28.50 ± 14.12	
Clinical classification		
Localized	23.00 ± 8.49	0.306
Generalized	23.39 ± 15.01	
Cephalic	40.00 ± 16.97	
The severity of tetanus		
Mild	16.00 ± 7.81	0.001^∗^
Moderate	20.53 ± 12.27	
Severe and very severe	29.64 ± 16.86	
Autonomic nervous dysfunction		
Yes	26.70 ± 17.27	0.081
No	20.76 ± 11.66	
Complications		
Yes	27.79 ± 16.01	0.001^∗^
No	15.85 ± 8.49	
Dose of TAT ≥ 30,000 UI/d (*N* = 75)		
Yes	24.43 ± 15.11	0.784
No	23.23 ± 15.73	
Artificial airway method (*N* = 39)		
Endotracheal intubation	25.27 ± 15.22	0.092
Tracheotomy	34.50 ± 16.80	

**Table 5:** The factors influencing the length of stay in the ICU.

Variable	The length of stay in the ICU (days)	*P* value
Dose of TAT ≥ 30,000 UI/d (*N* = 56)		
Yes	20.74 ± 13.98	0.977
No	20.64 ± 12.40	
Artificial airway method (*N* = 39)		
Endotracheal intubation	17.47 ± 10.57	0.003
Tracheotomy	29.46 ± 11.97	

## Data Availability

The patients' data used to support the findings of this study are available from the corresponding author upon request.
